# Sexual consent norms in a cross-sectional national sample of the UK

**DOI:** 10.1093/pubmed/fdab361

**Published:** 2021-10-16

**Authors:** Malachi Willis, Tiffany L Marcantonio

**Affiliations:** Institute of Health and Wellbeing, University of Glasgow, Glasgow G12 8QQ, UK; Department of Health, Human Performance, and Recreation, University of Arkansas, Fayetteville, AR 72701, USA; Kinsey Institute for Research in Sex, Gender, and Reproduction, Bloomington, IN 47405, USA

**Keywords:** sexual consent, sexual assault, affirmative consent, sexual refusal, gender

## Abstract

**Background:**

Sexual assault is a pervasive problem in the UK, and young women are disproportionately affected. We sought to provide an initial account of sexual consent norms in the UK and whether they differ by gender and age.

**Method:**

The present study was a secondary analysis of data collected by the Family Planning Association, which conducted an online survey (*N* = 2003) to assess experiences with, knowledge of, and attitudes toward consent. The sample represented all regions of the UK and spanned ages 14–55.

**Results:**

Definitions of sexual consent endorsed by women and older age groups more closely aligned with the tenets of affirmative consent compared with men and younger age groups. Women and older age groups were also more likely to perceive that various nonverbal cues may be used to interpret sexual consent or refusal and were more supportive of people being able to withdraw their sexual consent.

**Conclusion:**

Maladaptive sexual consent norms seemed to be prevalent among men and young people in the UK, which may contribute to young women’s elevated risk of experiencing sexual assault. Our findings support the UK’s recent relationships and sex education curriculum that actively promotes healthy sexual consent norms.

## Introduction

Sexual assault is a pervasive problem in the UK, and young women are disproportionately affected. In England and Wales, 3.4% of women aged 16–59 experienced sexual assault in the past year compared with 0.9% of men aged 16–59.^1^ Demonstrating the increased risk younger women experience, 69% of women who experienced rape or assault by penetration since the age of 16 reported that they were most recently raped when they were aged 16–24, 18% were aged 25–34, 10% were aged 35–44 and 3% were aged 45–54.^2^ Given these high prevalence rates, the negative effects of sexual assault on victims’ well-being (e.g. psychological trauma, anxiety/depression, substance use, physical harm)^3^^,^^4^ constitute a public health crisis in the UK.

Sexual assault and rape are legally defined as involving the absence of sexual consent.^5^ Therefore, population-level statistics on norms related to sexual consent may help understand the experiences people in the UK have regarding nonconsensual sexual activity. Further, assessing how sexual consent norms might vary by gender or age group may provide potential explanations for why young women are at increased risk—especially because the perpetrators of these sexual assaults are predominantly young men.^6^ Key aspects of sexual consent that would provide a foundation for capturing how people in the UK conceptualize consensual and nonconsensual sexual activity include norms for defining, communicating and withdrawing sexual consent.

In the UK, sexual consent is legally defined as ‘a person consents if [they agree] by choice, and has the freedom and capacity to make that choice’.^5^ A person’s willingness to agree to sexual activity can vary across contexts like previously having had sex with the other person^7^ or using alcohol or other drugs.^8^ Thus, sexual consent is fluid and may change from one moment to the next. Women tend to define sexual consent as a process, whereas men are more likely to perceive sexual consent as a one-time sexual event.^9^ Regarding age, a recent study found that, at a basic level, sexual consent is similarly defined across the adult lifespan as agreement to engage in sexual activity.^10^

More nuanced definitions of sexual consent might also include the verbal or nonverbal communication of willingness to engage in sexual activity.^11^^,^^12^ Definitions of affirmative consent, which have become the standard for many sexual consent education curricula (e.g. ‘yes means yes’), suggest that this communication be enthusiastic and explicit—even though evidence suggests that people are able to effectively use and interpret implicit or subtle consent communication.^13^^,^^14^ In any case, formal definitions typically no longer accept the absence of a refusal as a sufficient indicator of sexual consent (e.g. ‘no means no’); indeed, passive communication cues like not responding do not reliably reflect people’s internal willingness^15^ and may even represent refusal.^16^ As such, the cues people think may be used to convey their own willingness to engage in sexual activity or infer that of others can shed light on norms related to this external aspect of sexual consent. Based on stereotypically gendered roles that promote the sexual agency of men, women tend to report greater reliance on nonverbal sexual consent cues than men.^17^^,^^18^ Further, younger adults are more likely than middle-aged or older adults to include explicit communication in the scope of their definition of sexual consent but less likely to include implicit communication.^10^

In addition to communication cues, people perceive that context cues may be used to infer another person’s willingness to engage in sexual activity. Contexts for which people might assume sexual consent include having engaged in sexual activity with each other before, drinking alcohol together and being in a bedroom or private setting.^7^^,^^9^ While these contexts may increase the probability that a person is willing to engage in partnered sexual activity, perceiving them to be absolute indicators of sexual consent can increase the risk of sexual assault.^12^ People must be able to freely withdraw their sexual consent disregarding the circumstances. Research shows that women and men may similarly understand sexual refusals,^16^ but men may be less supportive of the right to withdraw sexual consent given their propensity to conceptualize sexual consent as a one-time event.^9^ Further, similar proportions of people across the adult lifespan included the retractability of consent in their definition.^10^

Understanding these sexual consent norms can help address sexual assault in the UK. In an initial assessment of sexual consent norms in the UK, a recent qualitative study found that university students defined sexual consent as an agreement that should ideally be explicit; however, their own experiences with sexual consent tended to rely on tacit understandings and subtle communication.^19^ Participants in that study additionally described how people can refuse sexual activity verbally or nonverbally and that communicating willingness to engage in sexual activity in one context does not necessarily translate to sexual consent in the future.^19^ Extending an empirical and quantitative focus on sexual consent to the UK—especially across diverse age groups—will help provide evidence for sexual health education to consider when aiming to reduce rates of sexual assault. Thus, we sought to describe norms in the UK regarding defining, communicating and withdrawing sexual consent.

## Method

### Procedure and sample

This was a secondary analysis of data collected by the Family Planning Association (FPA). For Sexual Health Week in 2018, FPA conducted an online survey of 2,003 people to assess experiences with, knowledge of and attitudes toward consent. Data were collected between 24 and 29 August 2018. The sample represented all regions of the UK and spanned ages 14–55 (Table 1). Further details regarding the FPA’s measure development, study protocol or recruitment strategies are unavailable.

**Table 1:** Sample characteristics

	*Total (N = 2003)*	*Women (n = 1039)*	*Men (n = 964)*
Age			
14–24	717 (36.0%)	361 (34.7%)	356 (36.9%)
25–34	422 (21.1)	173 (16.7)	249 (25.8)
35–44	379 (18.9)	211 (20.3)	168 (17.4)
45–54	485 (24.2)	294 (28.3)	191 (19.8)
Region			
East Midlands	125 (6.2)	75 (7.2)	50 (5.2)
East/East Anglia	143 (7.1)	86 (8.3)	57 (5.9)
London	482 (24.1)	149 (14.3)	333 (34.5)
North East	141 (7.0)	93 (9.0)	48 (5.0)
North West	208 (10.4)	122 (11.7)	86 (8.9)
Northern Ireland	36 (1.8)	16 (1.5)	20 (2.1)
Scotland	131 (6.5)	60 (5.8)	71 (7.4)
South East	211 (10.5)	124 (11.9)	87 (9.0)
South West	133 (6.6)	80 (7.7)	53 (5.5)
Wales	80 (4.0)	47 (4.5)	33 (3.4)
West Midlands	176 (8.8)	104 (10.0)	72 (7.5)
Yorkshire/Humber	137 (6.8)	83 (8.0)	54 (5.6)

No other sample characteristics were provided in the secondary dataset.

### Patient and public involvement

Data from individual participants are not accessible for this secondary analysis; so no participants or the public were involved in the present study.

### Measures

The survey included items regarding three aspects of sexual consent norms. First, an item assessed people’s definitions of sexual consent: ‘What do you think consent for sexual activities means?’ A second item asked about nonverbal communication cues that may be used to infer a person’s sexual consent, ‘If someone didn’t say yes or no verbally, which of these ways could you tell how they feel?’ Finally, two items assessed perceived norms for withdrawing sexual consent. Responding on a five-point scale from ‘No, never’ to ‘Yes, always’, participants were asked, ‘Do you think it is OK for people to change their mind about consent while engaging in sexual acts?’ They then selected all that applied from a list of contexts in response to ‘Is it OK for somewhat to withdraw consent if…’ Response options for each item are provided in Tables 2 and 3.

**Table 2:** Sexual consent variables by gender

	*Total (N = 2003)*	*Women (n = 1039)*	*Men (n = 964)*	χ^2^	*P*	φ_C_
Defining sexual consent						
A clear ‘yes’ is needed every single time	1149 (57.4)	647 (62.3)	502 (52.1)	21.26^*^	<0.001	0.103
Someone doesn’t have to ask their partner if they’ve said yes before	229 (11.4)	85 (8.2)	144 (14.9)	22.55^*^	<0.001	0.106
Figuring it out from how they act	293 (14.6)	114 (11.0)	179 (18.6)	23.11^*^	<0.001	0.107
Someone’s ‘yes’ still counts even when they are very intoxicated	347 (17.3)	129 (12.4)	218 (22.6)	36.31^*^	<0.001	0.135
There’s no need to ask if the people fancy each other	229 (11.4)	81 (7.8)	148 (15.4)	28.20^*^	<0.001	0.119
When someone says ‘yes’ to what they want or ‘no’ to what they don’t	1012 (50.5)	536 (51.6)	476 (49.4)	0.98	0.323	0.022
If a person doesn’t say ‘no’	242 (12.1)	94 (9.0)	148 (15.4)	18.72^*^	<0.001	0.097
Communicating sexual consent						
Eye contact	685 (34.2)	379 (36.5)	306 (31.7)	4.98^*^	0.026	0.050
Moving closer or further away	1036 (51.7)	594 (57.2)	442 (45.9)	25.66^*^	<0.001	0.113
How comfortable they are undressed	652 (32.6)	326 (31.4)	326 (33.8)	1.36	0.244	0.026
Whether they initiate contact	963 (48.1)	485 (46.7)	478 (49.6)	1.69	0.193	0.029
Their facial expression	903 (45.1)	500 (48.1)	403 (41.8)	8.06^*^	0.005	0.063
If someone goes quiet or is more talkative	681 (34.0)	381 (36.7)	300 (31.1)	6.86^*^	0.009	0.059
Withdrawing sexual consent						
If they have been bought dinner	1086 (54.2)	624 (60.1)	462 (47.9)	30.16^*^	<0.001	0.123
If they have been bought drinks	1281 (64.0)	700 (67.4)	581 (60.3)	10.94^*^	<0.001	0.074
If they have already kissed the other person	1152 (57.5)	647 (62.3)	505 (52.4)	20.00^*^	<0.001	0.100
If they are in a bedroom	1095 (54.7)	625 (60.2)	470 (48.8)	26.22^*^	<0.001	0.114
If they have had sex with that person before	1030 (51.4)	600 (57.7)	430 (44.6)	34.57^*^	<0.001	0.131
If they are already naked	944 (47.1)	569 (54.8)	375 (38.9)	50.50^*^	<0.001	0.159

For each item, participants were asked to select all response options that applied; thus, percentages sum to values >100.

^*^This χ^2^-value remained significant (α = 0.05) once adjusting the *P*-value according to Benjamini and Hochberg’s (1995) procedure for controlling the false discovery rate.

**Table 3:** Sexual consent variables by age group

	*14–24 (n = 717)*	*25–34 (n = 422)*	*35–44 (n = 379)*	*45–54 (n = 485)*	χ^2^	*P*	φ_C_
Defining sexual consent							
A clear ‘yes’ is needed every single time	387 (54.0)	205 (48.6)	228 (60.2)	329 (67.8)	39.64^*^	<0.001	0.141
Someone doesn’t have to ask their partner if they’ve said yes before	95 (13.2)	92 (21.8)	29 (7.7)	13 (2.7)	89.18^*^	<0.001	0.211
Figuring it out from how they act	111 (15.5)	100 (23.7)	42 (11.1)	40 (8.2)	47.84^*^	<0.001	0.155
Someone’s ‘yes’ still counts even when they are very intoxicated	145 (20.2)	124 (29.4)	45 (11.9)	33 (6.8)	92.40^*^	<0.001	0.215
There’s no need to ask if the people fancy each other	96 (13.4)	98 (23.2)	23 (6.1)	12 (2.5)	109.85^*^	<0.001	0.234
When someone says ‘yes’ to what they want or ‘no’ to what they don’t	335 (46.7)	202 (47.9)	197 (52.0)	278 (57.3)	14.62^*^	0.002	0.085
If a person doesn’t say ‘no’	90 (12.6)	84 (19.9)	31 (8.2)	37 (7.6)	38.95^*^	<0.001	0.139
Communicating sexual consent							
Eye contact	213 (29.7)	143 (33.9)	131 (34.6)	198 (40.8)	15.93^*^	0.001	0.089
Moving closer or further away	334 (46.6)	171 (40.5)	226 (59.6)	205 (62.9)	62.49^*^	<0.001	0.177
How comfortable they are undressed	240 (33.5)	125 (29.6)	122 (32.2)	165 (34.0)	2.43	0.489	0.035
Whether they initiate contact	295 (41.1)	201 (47.6)	188 (49.6)	279 (57.5)	31.54^*^	<0.001	0.125
Their facial expression	303 (42.3)	170 (40.3)	193 (50.9)	237 (48.9)	14.26^*^	0.003	0.084
If someone goes quiet or is more talkative	237 (33.1)	133 (31.5)	131 (34.6)	180 (37.1)	3.59	0.309	0.042
Withdrawing sexual consent							
If they have been bought dinner	335 (46.7)	183 (43.4)	238 (62.8)	330 (68.0)	84.83^*^	<0.001	0.206
If they have been bought drinks	419 (58.4)	282 (66.8)	246 (64.9)	334 (68.9)	16.20^*^	0.001	0.090
If they have already kissed the other person	377 (52.6)	203 (48.1)	235 (62.0)	337 (69.5)	54.00^*^	<0.001	0.164
If they are in a bedroom	352 (49.1)	162 (38.4)	237 (62.5)	344 (70.9)	115.32^*^	<0.001	0.240
If they have had sex with that person before	315 (43.9)	143 (33.9)	229 (60.4)	343 (70.7)	152.66^*^	<0.001	0.276
If they are already naked	279 (38.9)	116 (27.5)	215 (56.7)	334 (68.9)	190.74^*^	<0.001	0.309

For each item, participants were asked to select all response options that applied; thus, percentages sum to values >100.

^*^This χ^2^-value remained significant (α = 0.05) once adjusting the *P*-value according to Benjamini and Hochberg’s (1995) procedure for controlling the false discovery rate.

### Analysis

To test whether each sexual consent variable was associated with gender or age, we conducted chi-squared tests of independence. Gender was a dichotomous variable (i.e. woman or man), and age was categorical (i.e. 14–24, 25–34, 35–44, 45–54 years). To account for inflated type-1 errors due multiple tests, we adjusted significance thresholds for *P*-values to control the false discovery rate (α = 0.05).^20^ We reported Cramér’s V (φ_C_) as a measure of effect size for the chi-squared tests. A φ_C_-value of 0.10 indicates a small effect size, 0.30 medium and 0.50 large.

## Results

### Defining sexual consent

The most commonly endorsed definition of sexual consent was ‘A clear “yes” is needed every single time’, which 57.4% of participants agreed with. At 11.4%, the least frequently endorsed definition was ‘Someone doesn’t have to ask their partner if they’ve said yes before’.

Across the seven definitions, there were six significant gender differences (Table 2). The largest gender difference was that 22.6% of men endorsed the definition that ‘someone’s “yes” still counts even if they are very intoxicated (e.g. high or drunk)’ compared with 12.4% of women. For each significant gender difference, women’s definitions of sexual consent more closely aligned with the tenets of affirmative consent.

There were significant age differences for all seven definitions (Table 3). Of note, 23.2% of participants aged 25–34 reported that ‘there’s no need to ask if the people fancy each other’ compared with 2.5% of participants aged 45–54. Across all definitions, greater proportions of the two older age groups endorsed tenets of affirmative consent than the two younger age groups.

### Communicating sexual consent

The most commonly endorsed nonverbal communication cue was positioning oneself (i.e. moving closer or further away), which 51.7% of participants reported being able to use to perceive sexual consent or refusal. Across the six nonverbal consent behaviors, there were four significant gender differences (Table 2). Proportionally more women than men indicated that they could infer a partner’s sexual consent based on positioning oneself (57.2% versus 45.9%), facial expressions (48.1% versus 41.8%), going quiet or being more talkative (36.7% versus 31.1%) and eye contact (36.5% versus 31.7%).

There were significant age differences for four of the six nonverbal consent behaviors (Table 3). Specifically, greater proportions of the two older age groups reported that they could use each of these nonverbal cues to infer sexual consent than the two younger age groups. The largest effect of age was for positioning oneself: 14–24 year olds (46.6%), 25–34 year olds (40.5%), 35–44 year olds (59.6%) and 45–54 year olds (62.9%).

### Withdrawing sexual consent

When asked about norms for withdrawing sexual consent, women (*M* = 4.55, standard deviation [SD] = 0.83) agreed more strongly than men (*M* = 3.95, SD = 1.09) that it is okay for people to change their mind about consent while engaging in sexual activity, *t*(780.66) = 9.88, *P* < 0.001, Cohen’s *d* = .633. Of note, 66.2% of women reported that withdrawing consent in the middle of a sexual act is ‘always’ okay—compared with 46.7% of men. Further, significantly fewer men than women indicated that it would be okay for someone to withdraw sexual consent across each of the six hypothetical contexts assessed (Table 2).

Age was also significantly associated with norms for withdrawing sexual consent, *F*(3, 1137) = 49.77, *P* < 0.001, *R*^2^ = 0.116. Pairwise comparisons indicated that participants aged 14–24 (*M* = 4.12) or aged 25–34 (*M* = 3.74) both endorsed lower levels of agreement that it is okay for people to change their mind about consent while engaging in sexual activity than those aged 35–44 (*M* = 4.49) or aged 45–54 (*M* = 4.68), *P*s < 0.001. Across all six contexts assessed, age groups significantly varied in the withdrawal norms they endorsed (Table 3). The oldest age group was the most supportive of people being able to withdraw their sexual consent.

## Discussion

### Main finding of this study

Using secondary data from a national sample of the UK, we found that men and younger age groups (i.e. 14–24, 25–34) were more likely than women or relatively older age groups (i.e. 35–44, 45–54) to endorse definitions that (i) assumed a person’s sexual consent if they have sexual history, (ii) permitted a person to be very intoxicated and (iii) interpreted the lack of refusal as consent. Such definitions of sexual consent are problematic and may contribute to young women’s elevated risk of experiencing nonconsensual sexual activity given that most sexual assaults are perpetrated by romantic partners,^21^ alcohol is involved in most sexual assaults^22^ and not refusing does not reliably reflect women’s willingness.^15^ Educational initiatives should discuss these misunderstandings of sexual consent that may contribute to a culture that places responsibility for sexual assault on women and consequently excuses men’s behaviours.

### What is already known on this topic

Extending previous evidence that women more commonly than men use nonverbal cues to communicate their sexual consent or interpret that of others,^17^^,^^18^ we found that women were also more likely to report that they can infer somebody’s willingness based on nonverbal cues like eye contact and facial expressions. Men’s diminished endorsement of these types of communication may result from being less familiar with using such implicit cues themselves, and younger people simply may not have the sexual experience of their older counterparts—restricting the array of potential consent cues they have been exposed to. For these reasons, sexual consent education initiatives should recognize that sexual consent communication is much more diverse than an explicit verbal enthusiastic yes. By unilaterally endorsing one type of cue, affirmative consent proponents deprive young people the opportunity to learn the many ways that people consent to or refuse sexual activity. Young men may perpetrate sexual assault if they are not able to competently and confidently interpret the implicit or nonverbal cues that are regularly used to communicate consent or refusal. Indeed, men who endorse passive rather than active consent communication have elevated odds of perpetrating coercive sexual assault.^23^ That said, miscommunication does not seem to be the underlying facilitator of many sexual assaults; rather, men may choose to ignore refusals that are not explicit and verbal—even though evidence suggests that they understand these cues as signs of unwillingness.^16^^,^^24^

Therefore, effective communication is not sufficient for sexual activity to be consensual; people also need to acknowledge and respect the times when potential partners withdraw their sexual consent or outright refuse sexual activity. Yet, in our sample, substantial proportions of people reported that it was not okay for somebody to withdraw their sexual consent across various contexts (e.g. having been bought drinks, having engaged in sexual activity before). Men and younger people were particularly likely to suggest that sexual consent should not be revoked under these circumstances, which may put younger women—who may not perceive their actions of accepting an alcohol beverage or kissing somebody as irrefutable indicators of their willingness to engage in further sexual activity—at risk of sexual assault.^9^^,^^12^

Our assessment of differences across age contributed to an academic literature that has primarily investigated sexual consent within university student populations.^15^ That we found relatively older age groups to endorse healthier sexual consent norms contradicts recent evidence that similar proportions of people across the adult lifespan focused on agreement to engage in sexual activity and retractability as core tenets of sexual consent.^10^ However, both studies suggest that relatively younger age groups are less likely to rely on implicit forms of consent communication. Young people’s stronger endorsement of explicit sexual consent cues may reflect the recent proliferation of sexual health education initiatives that emphasize affirmative consent.^13^

### What this study adds

Young men’s problematic views of sexual consent may contribute to the elevated risk of sexual assault among young women as well as gender or sexual minorities (e.g. nonbinary people, men who have sex with men). To promote healthy and consensual sexual encounters, prevention programmes should not only target young men’s maladaptive attitudes and behaviours regarding sexual consent but also aim to widely facilitate respect for all people’s sexual agency and empowerment to effectively communicate about their sexuality.

Overall, our findings support the UK’s recent development of a relationships and sex education curriculum that is now legally compulsory for secondary schools and must have been implemented by the summer term 2021 at the latest.^25^ Of note, parallels may be drawn from these data to provide empirical support for specific knowledge goals that this curriculum expects students to achieve by the end of secondary school (e.g. ‘how people can actively communicate and recognise consent from others, including sexual consent, and how and when consent can be withdrawn’). Such nuances regarding sexual consent norms were the focus of the present study, which provided evidence that young men in the UK are in particular need of effective sexual consent education. For those who have already completed secondary school, public health initiatives could consider promoting healthy sexual consent norms via social media campaigns, public service announcements or advertisements on online dating apps—all of which can be designed to target specific sociodemographic groups for increased efficacy.

### Limitations of this study

Many of the present study’s limitations were due to the data’s secondary nature, which prevented us from providing a comprehensive and critical account of the study protocol. For example, we did not have access to details regarding methodology or key sociodemographic characteristics of the sample (e.g. education level, partner status). Another limitation was that person-level data were not made available, which precluded us from separating the effects of gender and age on sexual consent norms or evaluating their potential interaction. These data were also limited in that they were cross-sectional, self-reported and retrospective—subjecting them to social desirability and recall biases. Further, the measures included in this study did not adequately delineate sexual consent versus sexual refusals, which involve separate but related communication processes.^16^ Despite these limitations, our preliminary findings from this national sample suggested that maladaptive sexual consent norms are prevalent among men and young people in the UK.

## Notes

**Malachi Willis,** PhD

**Tiffany L. Marcantonio,** MA
